# Epidemiologic parameters and evaluation of control measure for 2009 novel influenza a (H1N1) in Xiamen, Fujian Province, China

**DOI:** 10.1186/1743-422X-9-20

**Published:** 2012-01-17

**Authors:** Jinyu Shen, Jianjun Niu

**Affiliations:** 1 Xiamen Municipal Center for Disease Control and Prevention, Xiamen City, Fujian Province, People's Republic of China; 2 Xiamen Center for Disease Control and Prevention, 681-685 Shengguang Rd. Jimei District, Xiamen 361021, Fujian Province, People's Republic of China

**Keywords:** Novel influenza A (H1N1), Epidemiologic Parameter, Control Measure, Evaluation

## Abstract

**Background:**

Containment of influenza A H1N1 virus spread was implemented successfully in Xiamen, with large-scale inoculation to reduce morbidity. To identify beneficial elements and to guide decision-making in epidemic containment, we analyzed the epidemiologic parameters and evaluated the control measures.

**Method:**

We determined various parameters from laboratory-confirmed cases, including incubation period, duration of illness and reproductive number (R_0_), and evaluated the control measures.

**Results:**

There were1414 cases with dates of onset between June 14, 2009 and March 22, 2010. The incidence was 56.79/100,000, and mortality was 0.12/100,000. The incidence during the community epidemic phase was 6.23 times higher than in the containment phase. A total of 296,888 subjects were inoculated with domestic influenza H1N1 virus cleavage vaccine. An epidemic curve showed that vaccination in students cut the peak incidence of illness significantly. Men (relative risk (RR) = 1.30, 95% confidence interval (CI): 1.17-1.45) and persons aged 0-14 years were at greater risk of infection. The incidence increased with younger age (*χ*^2 ^= 950.675, *p *= ∞). Morbidity was lower in urban than in rural areas (RR = 0.56, 95%CI: 0.50-0.62). The median incubation time was 2 days, median duration of symptoms was 7 days, and the within-school reproductive number was 1.35.

**Conclusion:**

Our analysis indicated that the characteristics of this novel influenza virus were similar to those of seasonal influenza. The principle of "interception of imported cases" applied at Xiamen ports, and vaccination of students effectively limited the spread of the influenza pandemic and reduced the epidemic peak.

## Introduction

**Figure 1:**
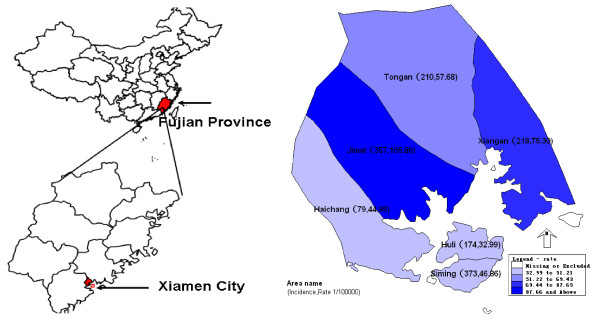
**Geographic location (in relation to China) and sketch map (based on EPIMAP) of Xiamen city (24.27N, 118.06E), Fujian Province, China**.

The emergence and global spread of the 2009 H1N1 influenza pandemic was a challenge to control in Xiamen city, Fujian Province, China. This is because the city is situated on the southeast coast of China (Figure 1), which is a special economic zone and sub-provincial area, whose planning is directly linked with central government, and it receives a large number of overseas visitors and holds various international conferences each year.

Our prevention and control strategy is different from other countries or regions. The containment strategy was implemented successfully in the early response days and large-scale inoculation with domestic influenza A H1N1 virus cleavage vaccine was conducted rapidly to reduce morbidity.

This surveillance data analysis aimed to understand the natural history of this novel influenza A H1N1 and to evaluate the control measures in Xiamen. Accurate determination of epidemiologic parameters can help guide future decision-making for novel infectious diseases. In particular, specific evaluation of control measures such as containment and vaccination against this novel influenza A H1N1 was very important to obtain useful information and experience in controlling an unknown respiratory pathogen.

## Materials and methods

A confirmed case was defined as a subject with laboratory-confirmed novel influenza A H1N1 virus infection by means of a real-time polymerase chain reaction (PCR) assay. We used data from the initial reports of laboratory-confirmed cases to determine the epidemiologic parameters of pandemic H1N1 influenza. The parameters included the incubation period, duration of illness and the reproductive number (R_0_). We evaluated the effectiveness of control measures using descriptive epidemiologic methods.

The process of containment was as follows. Suspected cases of fever (≥37.5°C) and acute respiratory symptoms were screened by the Xiamen Entry-Exit Inspection and Quarantine Bureau, and were transported to the Designated Medical Observational Station for additional testing. Nasopharyngeal and oropharyngeal specimens were collected with the swab technique from all suspect cases who had symptoms resembling those of seasonal influenza, including fever, cough, rhinorrhea, body aches, sore throat, and headache. Specimens were tested for influenza virus at the laboratory with the use of real-time PCR. All positive cases were interviewed to obtain demographic, epidemiologic and clinical information by epidemiologists of Xiamen Center for Disease Control and Prevention (CDC). All close contacts of cases were placed under 7 days' medical observation by the Community Health Service Centers. Of these, the transmission chain in 23 cases was very clear, and we collected data on their earliest exposure and onset of illness. An immediate epidemiological investigation was carried out by the Emergency Response Department of Xiamen CDC into all outbreaks. All information obtained was reported to the China Information System of Disease Control and Prevention.

We were granted access to the China Information System of Disease Control and Prevention data, which included all confirmed cases. We analyzed individual-level data on laboratory-confirmed cases of novel influenza A H1N1 in the province of Fujian, Xiamen, with a reported date of symptom onset between June 14, 2009 and March 22, 2010. This cutoff was chosen because individual-level reporting of cases of pandemic H1N1 influenza in Xiamen was stopped after this date. For these cases, information was available on age, date of symptom onset and, for patients admitted to hospital, the date of admission and discharge. Demographic information was available from the Xiamen Municipal Statistics Bureau.

The median incubation period of the 2009 H1N1 influenza virus was calculated using the records of the transmission chain of the primary case and secondary cases by identifying date of exposure and the date of the onset of symptoms (the time between infection and the onset of symptoms) in persons with confirmed cases. The most likely date of exposure was estimated as the midpoint between the earliest and most recent dates of exposure.

The duration of illness was estimated from the dates of onset and recovery reported by persons with confirmed 2009 H1N1 influenza using methods similar to those used to evaluate the period of incubation.

In an outbreak in one school in Xiamen, we estimated the within-school reproductive number using the principle of exponential growth period to characterize transmission. This analysis was based on the rate of increase in the number of 2009 H1N1 influenza cases among the school students.

## Results

A total of 1414 laboratory-confirmed cases of 2009 H1N1 influenza had reported dates of symptom onset between June 14, 2009 and March 22, 2010. The incidence was 56.79/100,000 (1414/2,490,000). Three persons died (death rate: 0.12/100,000). During the containment phase (June 14-August 31, 2009), 91 cases (6.44%) were identified, and during the community epidemic phase (September 1, 2009-March 22, 2010), 1323 cases (93.56%) were identified, and eight outbreaks occurred. The mean number of cases per month during the community epidemic phase was 6.23 times higher than during the containment phase.

**Figure 2:**
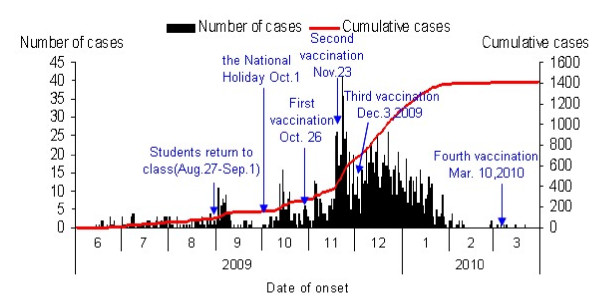
**Number of cases by date of onset (June 14, 2009, to March 22, 2010)**.

**Figure 3:**
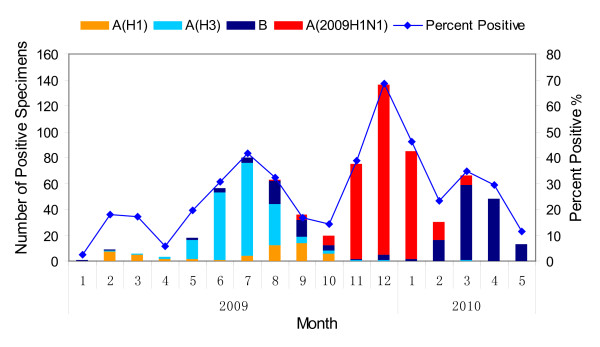
**Laboratory-confirmed cases of influenza (pandemic (H1N1) 2009 and seasonal) in Xiamen, 1 January, 2009-31 May, 2010, by month and type**.

The first case occurred on June 14, 2009. The number of new cases fluctuated at a very low level from June 14 to August 31, 2009. The first peak of the epidemic curve occurred on September 2, when students from all over the country returned to their colleges and universities. The second peak of the epidemic curve occurred after the National Holiday, and the cases gradually increased and reached the highest peak in mid-November (Figure 2). The first inoculation program with domestic influenza A H1N1 virus cleavage vaccine was launched among primary school and kindergarten teachers, civil servants and medical staff on October 26. The incidence did not significantly decrease. The second and third vaccination programs were carried out among students and teachers in universities, and primary and secondary schools, and the incidence began to drop (Table 1, Figure 2). A total of 296,888 persons were vaccinated. The epidemic curve showed that the control measures of containment and vaccination had a good effect (Figure 2). The decreasing trend of positive specimens in influenza surveillance (2009 H1N1 and seasonal influenza, by month and type) from 1 January 2009 to 31 May 2010 showed the first flu pandemic had ceased in Xiamen (Figure 3).

**Table 1:** Time of launch and number of inoculations for influenza A (H1N1) vaccinations

Start time of vaccination	First (October 26)	Second (November 23)	Third (December 3)	Fourth (March10)	Total
Target population	49500	57711	106188	83419	296818
Civil servant	19506	2841	-	-	22347
Medical staff	15418	-	-	-	15418
Primary school and kindergarten teacher	14515	-	-	-	14515
Senior school student	-	44144	-	-	44144
Junior school staff	-	10901	-	-	10901
Primary school student	-	-	43829	83419	127248
Junior school student	-	-	57307	-	57307
Other population	-	-	1085	3923	5008
Total	49439	57886	102221	87342	296888
Percentage of cumulative accomplishment %	99.9	100.3	96.3	104.7	100.0

**Figure 4:**
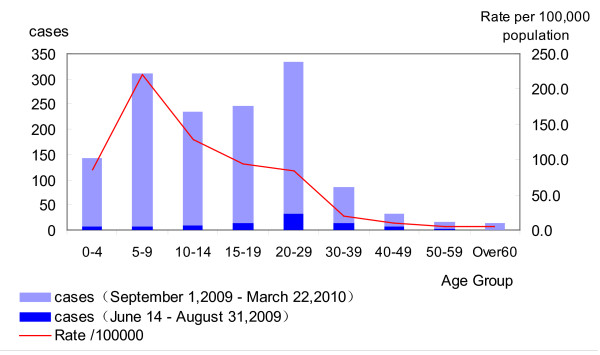
**Distribution of novel influenza A (H1N1) by age group, Xiamen, June 14, 2009-March 22, 2010**.

All age groups had reported laboratory-confirmed cases of novel influenza A (H1N1) virus. Of the 1414 cases, the mean age of the patients was 16.48 ± 0.31 years (standard deviation 11.66). The peak of morbidity was in the group of children aged 5-9 years old. The age distribution is presented in Figure 4. Men (relative risk (RR) = 1.30, 95% confidence interval (CI): 1.17-1.45) and persons aged 0-14 years were at greater risk of influenza A. The incidence gradually increased with younger age (*χ*^2 ^= 950.675, *p *= ∞). Deaths occurred among those aged > 50 years (Table 2, Figure 4). Overall, 63.2% (893/1414) of cases were students. It is to be noted that the proportion of students rose dramatically, from 37.4% in the containment phase to 64.9% in the community epidemic phase (Table 3); 51.63% (730/1414) of cases were reported from the Siming and Jimei districts, and 46.5% (327/703) of schools and colleges are located in these two districts. The morbidity in urban areas was lower than in rural areas (RR = 0.56, 95% CI: 0.50-0.62) (Figure 1).

**Table 2:** Confirmed cases by age group and sex--Xiamen City, June 14, 2009-March 22, 2010

Age group	Male (Deaths)	Rate/100000	Female (Deaths)	Rate/100000	Total (Deaths)	Rate/100000	RR (95% CI)
0-4	90	102.98	53	65.69	143	85.08	1.02 (0.83-1.24)
5-9	182	245.28	128	192.53	310	220.35	2.63 (2.25-3.08)
10-14	138	139.25	97	115.25	235	128.23	1.53 (1.29-1.82)
15-19	168	120.91	78	62.28	246	93.12	1.11 (0.94-1.31)
20-29	162	79.44	171	88.38	333	83.79	Ref. 1.00
30-39	42	20.13	43	20.58	85	20.36	0.24 (0.19-0.31)
40-49	17	9.74	15	8.73	32	9.24	0.11 (0.08-0.16)
50-59	8	4.91	9 (1)	5.75	17 (1)	5.32	0.06 (0.04-0.11)
60-69	7 (1)	9.56	2	5.35	9 (1)	6.47	0.08 (0.04-0.15)
Over 70	3 (1)	12.55	1	5.22	4 (1)	7.34	0.09 (0.03-0.24)
Total	817 (2)	64.08	597 (1)	49.13	1414 (3)	56.79	Chi X^2 ^= 950.675, p∞

CI = confidence interval

**Table 3:** Confirmed cases by occupation--Xiamen City, June 14, 2009-March 22, 2010

Occupation	Jun.14-Aug.31, 2009		Sep.1, 2009-Mar.22, 2010		Total	
	
	N	%	N	%	N	%
Student	34	37.4	859	64.9	893	63.2
Various types of employee	35	38.5	138	10.4	173	12.2
Worker	5	5.5	109	8.2	114	8.1
Preschooler	5	5.5	78	5.9	83	5.9
Farmer	4	4.4	58	4.4	62	4.4
Unemployment	6	6.6	53	4.0	59	4.2
Teacher	2	2.2	17	1.3	19	1.3
Medical worker	0	0.0	11	0.8	11	0.8
Total	91	100.0	1323	100.0	1414	100.0

A total of 23 cases contained sufficient information on earliest exposure and illness onset to estimate incubation periods. The median time from exposure to symptom onset was 2 days (25-75 percentile, 1-3 days). The estimated incubation periods fitted a log-normal distribution (mean 2.33 days, 95% CI: 1.71-2.94 days). The longest incubation period for 2009 H1N1 influenza was defined as 5 days and the 95th percentile was 4.94 days.

All cases, including minor, intermediate and severe cases, were hospitalized to isolate the source of infection in the early response days. We selected 333 confirmed cases of 2009 H1N1 influenza with information recorded for hospital discharge and illness onset time. We estimated that the median duration of illness was 7 days (25-75 percentile, 6-9 days), and 95th percentile was 12 days. The shortest and longest duration of illness for 2009 H1N1 influenza was 3 days and 22 days, respectively. The median duration was longer among patients aged younger than 14 years (7.69 days) than among older patients (7.0 days) (*χ*^2 ^= 22.17, *p *= 0.046). Intervals between onset and resolution of symptoms followed a log-normal distribution (mean duration 7.63 days, 95% CI: 7.38-7.89 days).

**Figure 5:**
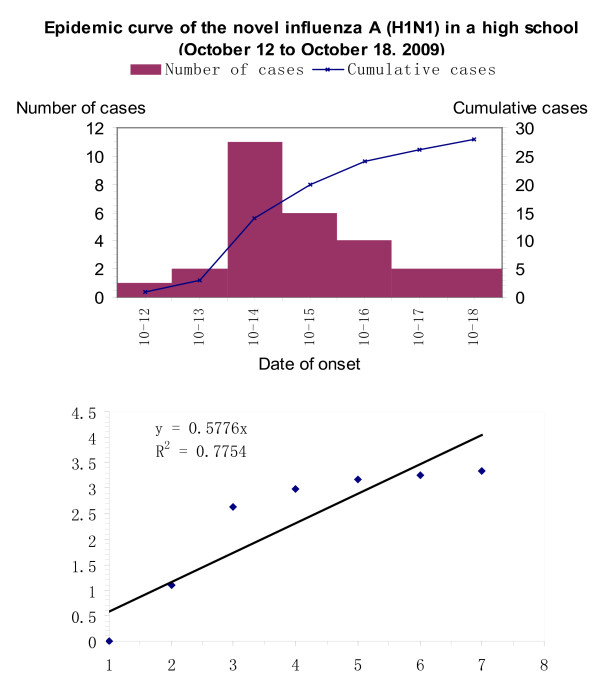
**Estimates of the within-school reproduction number of the novel influenza A virus (H1N1) infection in Xiamen**.

We estimated the reproductive number of the within-school outbreak at a high school from October 12 to October18. The transmission parameter of the fitted regression line was 0.5776, and multiplied by mean incubation (2.33 days) gave a within-school reproductive number of 1.35 (Figure 5). The formula is shown below. Y = e ^ht/the mean incubation^, according to the regression line y = 0.5776 t = ht/2.33, then h = 1.35.

## Discussion

As the pandemic spread, we strictly implemented the containment strategy in the early days to mitigate and control disease in the face of uncertainty of the vector. This period, defined as the "containment" phase, ranged from May 1 to August 31, 2009, and included body temperature screening and administration of health questionnaires at international ports of entry, isolation of infected travelers, and quarantine of close contacts. There was no outbreak in the containment phase, and the morbidity was far less than that of the community epidemic phase. It proved that implementation of a containment strategy can significantly decrease morbidity and mortality in the early stage. This was a necessary measure based on considerations of public health ethics, because we could not predict whether the virulence would increase in the future.

When all schools reopened in September, an outbreak occurred in many schools and the incidence increased rapidly. This period was defined as "the community epidemic" phase and began on September 1, 2009. Influenza vaccination is still considered the best way to prevent the spread of influenza occurrence. The domestic influenza A H1N1 virus cleavage vaccine showed good safety and immunogenicity, and inoculation with one dose of 15 μg vaccine in a schedule of 0 and 21 days induced a satisfactory immune effect in the population aged 12-60 years [1]. On November 24, we sequentially conducted large-scale vaccine immunization four times to reduce morbidity and outbreaks in different groups of people. However, the epidemic curve showed only the second and the third vaccination in students had reduced the peak of the illness significantly. This indicated that students are a high-risk population and that preventive health education in schools should be strengthened. The seasonal peak of influenza in Xiamen has been from February to June for many years. The incidence curve (Figure 2) showed a significant decrease from February (after large-scale inoculation among students), indicating that vaccination aided in the reduction of new cases of influenza.

Our estimate of mean incubation period (time from infection to onset of symptoms) of 2.33 days was consistent with typical seasonal influenza [2]. Our estimate of the duration of symptoms (median, 7.63 days) was also compatible with that of seasonal influenza. The longest incubation period was defined as 5 days and the 95th percentile was 4.94 days. The recommend of two days shorten should be made for the close contacts under 7 days of medical observation theoretically. In contrast, our low estimate of 1.35 for the basic reproductive number (*R_0_*) was different from the estimate in the United States (1.7-1.8 after adjustment for increasing ascertainment of cases) [3]. Our *R_0 _*estimate is a range determined within schools. The reproduction number is usually calculated using a longer period of incidence data and it can be affected by many factors. We chose a school outbreak to calculate the reproduction number for two main reasons. First, the outbreak occurring in a school signified containment failure and the spread of H1N1 in the community. Second, vaccination had not been carried out when the outbreak was first reported. Therefore, the calculation of the reproduction number should be closest to the true value in this natural state of viral spread.

The limitation of our analysis was that the laboratory-confirmed cases we analyzed almost certainly represented a small subset of cases of pandemic H1N1 influenza during the period as only laboratory-confirmed cases were analyzed [4]. We did not include infected subjects without symptoms or those with symptoms who did not undergo laboratory testing. The proportion of infected people who were actually identified as cases was needed to estimate the true morbidity and mortality during the community epidemic phase. However, these are likely to be representative because of the strict screening and management of suspect cases of fever during the containment phase.

## Conclusion

On the basis of data from initial laboratory-confirmed cases of pandemic H1N1 influenza, our estimates show that the characteristics of this novel influenza virus were similar to those of seasonal influenza. Our relatively low estimate of *R_0 _*indicates that effective use of containment strategies may substantially reduce the eventual size of the pandemic. A series of correct strategies for prevention and control achieved good results. These measures included the principle of "interception of imported cases" applied at Xiamen ports, and vaccination of students which effectively attenuated the spread of the influenza pandemic and reduced the epidemic peak.

## Abbreviations

RR: Relative risk; CI: Confidence interval; PCR: Polymerase chain reaction; CDC: Center for Disease Control and Prevention; R_0_: Reproductive number.

## Competing interests

The authors declare that they have no competing interests.

## Authors' contributions

JS collected the data, performed the statistical analysis and interpretation. In addition, she prepared and advised the manuscript. JN participated in collection of data. All authors read and approved the final manuscript.

## Authors' information

Niu Jianjun: male, Chief Technician, Professor, Tutor for graduate, Director of Xiamen Center for Disease Control and Prevention (XMCDC), dedicated to infectious disease control, studying on pathogen, microbiology and molecular epidemiology. Member of American Society for Virology, Executive member of National Association for the Prevention of Tuberculosis (NAPT), Standing member of Fujian Epidemiology and Hygiene committee, Vice-president of Xiamen Prevention Medicine Association; Corresponding Editor of *Chinese Journal of Preventive Medicine*, member of editorial board of *Occupation and Health *and *Strait Journal of Preventive Medicine*, Supervisor of China-U.S.A. birth cohort study and several programs funded by Chinese Ministry of Health Research Fund and Fujian Natural Science Foundation; Main participant of *the National Eleventh Five-Year Plan *key program for prevention of severe contagious diseases such as AIDS and virus hepatitis supported by the Ministry of Sci-tech of the PRC, National Science Supported Programs and Programs funded by National Natural Science Fund. Visiting scholar to the Australian National University in 2005 and to the Public Health School, Yale University in 2009.
